# Palliative Farming

**DOI:** 10.1007/s10892-022-09404-7

**Published:** 2022-09-13

**Authors:** Ole Martin Moen, Katrien Devolder

**Affiliations:** 1 Faculty of Health Sciences, Oslo Metropolitan University, Box 4 St. Olavs plass, 0130 Oslo, Norway; 2 Faculty of Philosophy, University of Oxford, Oxford Uehiro Centre for Practical Ethics, Suite 8, Littlegate House, St Ebbes Street, Oxford, OX1 1PT UK

**Keywords:** Animal ethics, Factory farming, Meat, Pharmacology, Veterinary ethics

## Abstract

Billions of animals live and die under deplorable conditions in factory farms. Despite significant efforts to reduce human consumption of animal products and to encourage more humane farming practices, the number of factory-farmed animals is nevertheless on an upward trajectory. In this paper, we suggest that the high levels of suffering combined with short life-expectancies make the situation of many factory-farmed animals relevantly similar to that of palliative patients. Building on this, we discuss the radical option of seeking to reduce the suffering of factory-farmed animals through the use of drugs that alleviate pain and distress, administered under a regimen where experiential wellbeing is prioritized over the aim of avoiding drug-dependence.

## Introduction

In order to meet worldwide demand for animal products, approximately 30 billion land animals are farmed and killed every year.^1^ More than 70% of these live in industrial farms, often referred to as *factory farms* (Anthis and Anthis 2019).

Factory farming poses a serious threat to animal welfare. Living in a factory farm often involves severe restrictions on movement and frustration of the animals’ instincts. Often, it also involves painful procedures such as castration, hot iron branding, beak trimming, tail docking, and horn removal (for a balanced overview, see Thompson 2020). In many cases, these procedures are performed without any form of pain relief (USDA et al. 2018).^2^

Human consumption of animal products is a topic of heated debate among ethicists. Although some ethicists, such as Christopher Belsaw (2016), J. Baird Callicott (2016), and Roger Scruton (2000), defend the practice—and Nick Zangwill (2021) argues that, under certain conditions, we have a moral duty to eat meat—it is noteworthy that these theorists all restrict their defenses to the consumption of animal products that originate from high quality free-range farms, or from hunting. Dan Shahar (2021) argues that it can be permissible to eat meat even if it comes from a factory farm, but this is not because factory farming is morally unproblematic; rather, Shahar suggests, it can be permissible insofar as one, instead of being an activist about animal welfare, is an activist about another issue that is at least equally important. To our knowledge, the only contemporary ethicist who outright defends factory farming is Timothy Hsiao (2017). Hsiao defends the practice by appealing to the premise that animal suffering is *morally irrelevant*. It is chilling that, among ethicists, this is the only ground on which our most common practice of treating animals is judged to be generally permissible.

We start by sketching what we take to be the central reason to believe that factory farming is a major moral problem, namely that it involves the suffering of a very large number of animals. We then go on to consider the main strategies that are currently employed to try to mitigate this problem, and argue that, though we support them, they are insufficient.

We subsequently present a *pro tanto* case for exploring another strategy. This strategy is motivated by the observation that, due to the high levels of suffering and the short life-expectancies in many factory-farmed animals, their situation has important features in common with that of palliative patients. The strategy that we consider is to give factory-farmed animals that share these features drugs that alleviate pain and distress under a regimen that prioritizes their experiential wellbeing over drug-dependence. We shall refer to farming practices in which such strategies are employed as *palliative farming*.

After introducing this strategy, we consider two categories of objections to further exploring it: *principled* and *pragmatic* objections. According to the principled the objections, palliative farming would be wrong regardless of its feasibility. According to the pragmatic objections, we shouldn’t explore palliative farming, because, even if it were acceptable in theory, there are so many practical problems with it, that we should not invest any time and effort in exploring it.

We argue that none of these objections provide a decisive reason against further exploring the ethics and feasibility of palliative farming. It is precisely because it is an open question whether palliative farms could be better for billions of animals compared to the *status quo* that we should invest time and effort into further exploring it.

## Why Factory Farming is a Major Moral Problem

The problem with which we are concerned in this paper is a commonly discussed one in the animal ethics literature: that of factory farming. Here is a simple, three-premise argument for why factory farming involves a major moral problem:

P1 : Factory farming involves the brutal treatment of billions of animals.

P2 : Animals tend to suffer when treated brutally.

P3 : The suffering of billions of animals is a major moral problem.

C : Factory farming involves a major moral problem.

The conclusion of this argument follows from its premises, so if one wants to reject the conclusion, one must reject at least one of its premises. Although our aim here is not to enter a detailed discussion of the nature of the problem, we would like to briefly explain why we think that none of the premises is easy to reject.

*Rejecting P1:* In order to reject P1, it might be suggested that most animals in factory farms are treated fairly well. Most farmers care about their animals, and animals in factory farms tend to be well fed, kept warm, and protected from predators.

Though there are indeed factory farmed animals that are treated fairly well (Thompson 2020), it would be naïve to assume that this is the norm. Consider the treatment of chickens, which account for the majority of factory farmed animals, roughly 24 billion individuals at any point in time (Statista 2020). Egg-laying chickens in factory farms are often crammed together in overcrowded sheds or wired cages where they cannot spread their wings, scratch, or dust-bathe, and they are bred to lay eggs at a very high frequency. Broiler chickens, raised for meat, tend to have more space, but they are bred to be constantly hungry and to grow so fast that their legs cannot support their weight. When slaughtered, moreover, many broiler chickens are grabbed by their legs (either by a human or machine) and hung upside down, which often results in broken legs, and if all goes well, they die when their heads are dipped in electrified water before their throats are slit (Humane Society 2009). If such practices do not qualify as brutal treatment, it is difficult to see what kind of treatment could qualify.

Importantly, P1 in the argument above does not state that *all*, or even that *most*, animals in factory farms are treated brutally; the premise is true insofar as billions, or “merely” hundreds of millions, of them are. Even if only a fraction of the billions of factory-farmed chickens that are subjected to such brutal treatment, the total number of chickens subjected to brutal treatment will still be very high.

We also want to emphasize that to hold P1 is not to hold that farmers *intend* a brutal treatment of animals. Well-intentioned farmers can have a large number of animals that suffer. Farmers typically have a very large number of animals to attend to and will make their living in a competitive system in which choosing to spend resources on improving the animals’ welfare beyond what is profitable will confer an economic disadvantage vis-à-vis competitors.

*Rejecting P2:* What about rejecting P2, i.e., denying that animals tend to suffer when treated brutally? It might be suggested that even though animals show aversive behavioral responses to noxious stimuli (e.g., trying to get away if touched with a hot poker), and such responses are occasioned by a first-person experience of suffering for us humans, it is unlikely that the aversive behavioral responses are also occasioned by a first-person experience of suffering in the case of animals. Is this plausible? We concede that such a view might be plausible given a religious worldview in which humans have originated separately from all other species. It coheres, however, neither with evolutionary biology nor with the findings of neuroscience. Birds and non-human mammals (which are the two classes of animals that we are primarily concerned with in this paper) have the same evolutionary origin as humans, and have the same pain pathways, react similarly to noxious stimuli, and stop reacting to them when anesthetized (Dawkins 2015). Since we and other animals are genetically, neurologically, and functionally very close, we would need weighty evidence to conclude that, despite these similarities, humans work in fundamentally different ways from other animals: humans consciously, animals non-consciously.

*Rejecting P3:* Finally, one can reject P3, i.e. deny that the suffering of billions of animals constitutes a major moral problem. One might, for example, argue that animal suffering is morally irrelevant, as Hsiao maintains, or that it is only negligibly relevant.

While we shall remain agnostic, in this paper, regarding the precise extent to which animal suffering matters, we shall assume that it matters sufficiently for the suffering of billions of animals to count as a major moral problem. Our argument does not assume that suffering is the *only* thing that matters morally; only that it is one of the things that matters to a significant extent.

To sum up, we suggest that to reject the conclusion that factory farming involves a major moral problem, one must either endorse a naïve empirical view about what is going on in factory farms (*rejecting P1*), endorse an implausible view about the mental capacities of animals (*rejecting P2*), or endorse a callous ethical view (*rejecting P3*).

## Current Strategies for Mitigating the Problem

We can distinguish between three main types of strategies for mitigating the moral problem of animal suffering brought about by factory farming.

*Reducing the number of animals:* One strategy for reducing the suffering brought about by factory farming is to reduce the number of animals that are factory farmed. This can be done by reducing demand for animal products from factory farms, for example, by promoting a plant-based eating, by promoting alternative sources of meat (such as ‘clean meat’ produced in vitro from stem cells, or animal products from organic, pasture-based farms), and/or by placing a higher tax on animal products that originate in factory farms.

While we support this strategy, we think that it is insufficient. Clean meat offers a valuable alternative to factory farmed meat, but it is likely to take decades for it to become widely available and affordable. Moreover, despite the growing popularity of plant-based alternatives to meat, especially in high-income countries, the world-wide number of factory-farmed animals is *growing*, and is expected to continue to grow over the coming decades, especially in lower- and middle-income countries (FAO 2012). As Thompson (2020: 24) points out, moreover, “veganism does nothing for the animals that continue to be housed in industrial conditions”.

*Mandating better living-conditions:* Another strategy—which can go hand in hand with the first—is to improve the living conditions of factory farmed animals, for example, by influencing consumer behaviors, and through legislation (e.g., the European Union’s ban on veal crates, European Union 2008), or other forms of regulations, including self-regulation by corporations, such as McDonalds and Walmart’s pledge to purchase only from suppliers using cage-free and crate-free animal housing systems (Shields et al. 2017).

Although living conditions for factory farmed animals have improved in some jurisdictions, the legal requirements for improvement are often minimal, including in the EU, and are also often breached. In the USA, where many states expressly exempt farm animals from anti-cruelty provisions, improvement is even slower (Animal Welfare Institute 2018). In some countries, including China, practices that have been phased out in other nations for animal welfare reasons remain commonplace, including battery cages for egg-laying chickens, sow stalls, and teeth trimming for pigs (Sinclair et al. 2020).

*Modifying the animals*: A third strategy to improve animal welfare in factory farms is to modify the animals themselves. Some routine practices fall within this category, such as tail-docking pigs for the sake of reducing injuries and infections (Edwards and Bennett 2014). Tail docking is controversial, however, because it is an invasive and painful strategy. Modifying animals to better adapt them to their environment has been dubbed animal *dis-enhancement* (Thompson 2008). One controversial example of animal dis-enhancement that could, possibly, nevertheless promote animal welfare is the use of genetic selection to produce blind chickens that engage less in pecking and cannibalism compared to sighted chickens (Sandøe et al. 2014). Recently, gene editing has been used to produce hornless dairy cows (horns cause injuries) and pigs resistant to deadly diseases (Burkard et al. 2018). Another type of genetic intervention aimed at improving animal welfare, the exploration of which is encouraged by Rollin (1995), is intervention into the genetic bases of animals’ reactions to stressful situations with the aim of breeding animals that are less frustrated by living in cages. In a similar vein, Shriver (2009) has argued that we should use genetic modification to produce livestock with a reduced capacity to suffer, by genetically modifying the animals to lack the enzymes in the brain responsible for enabling the affective dimension of chronic or persistent pain.

The types of genetic modification discussed by Rolling and Shiver, however, are currently not available, and if or when they become available, they are likely to be costly.

What can we do in the meantime?

## A *Pro Tanto* Case for Palliative Farming

When we are considering the range of possible solutions to the moral problem of factory farming, it is worth noting that insofar as we hold that a major part of the problem lies specifically in the *suffering*, the immediate cause of the problem is a *process in the neuronal systems of the animals*. In that case, we should ask if there could be ways to reduce suffering among factory-farmed animals, not only by means of the above strategies, but also by means of directly targeting the neuronal processes of animals in factory farms.

One common strategy for combating suffering is to administer drugs that alleviate pain and distress, such as benzodiazepines, opiates, and non-steroidal anti-inflammatory drugs (NSAIDs). Many drugs in these categories have been studied and used over several decades, both in human and in veterinary medicine.

When drugs that alleviate pain and distress are used, whether in animals or humans, they are typically used restrictively, so as to avoid drug dependence and other negative long-term effects. We find a notable exception to this restrictive approach, however, in *palliative* medicine. Since patients in palliative care have a short remaining life-expectancy (typically less than a year), the aim of palliative care is to alleviate suffering and promote quality of life in whatever time remains. For this reason, palliative patients are given gradually larger doses of drugs, and are allowed to die in a drug-dependent state. While it is important, also in palliative care, to avoid building drug tolerance too quickly, and to reduce the negative side-effects from drugs, the patients’ experiential wellbeing is given priority over concerns about drug dependence because the patient will not live long enough to experience the withdrawals.

We want to suggest that many animals in factory farms are in situations that warrant a palliative approach to decision-making. The reason is that, like palliative patients, many animals in factory farms, in addition to experiencing high levels of pain and distress, also have short life expectancies. The clearest example is broiler chickens, which, in factory farms, typically have a total lifespan of less than six weeks. (Normally, chickens can live for 5+ years.)

Could factory-farmed animals that suffer significantly be administered drugs under a palliative regimen where they can receive increasing doses of drugs that alleviate pain and distress? If so, it seems that this could be a way by which millions—or, possibly, billions—of animals lives that would otherwise be painful and distressing. Given the high number of animals that suffer in factory farms, this type of approach could, theoretically, constitute a *major* improvement compared to the *status quo*.

Although palliative farming (as we call it) might, at first glance, seem far-fetched and purely theoretical, two things should be noted. The first is that, since the early 2000s, palliative care for pets has become part of mainstream veterinary medicine (Goldberg 2016; Selter et al. 2022). The question that we are facing, therefore, is not whether it is possible to establish an entirely new practice, but whether it is possible to scale up (at least some aspects of) the already well-established practiced of palliative veterinary care.

The other thing that should be noted is that drugs such as benzodiazepines, opiates, and NSAIDs are already mass-produced, and can be manufactured at a relatively low cost. According to UNICEF Supply Division figures, the production cost, per standard human dose, is $0.014 for diazepam, the most common benzodiazepine, $0.065 for morphine, the most common opioid, and $0.009 for ibuprofen, the most common NSAID (Management Sciences for Health 2016: ‘Diazepam’, ‘Morphine’, ‘Ibuprofen’).

Admittedly the individualized treatment that is given in both human and veterinary palliative medicine, palliative farming can almost certainly not be individualized to the same extent, unless it is made extremely costly or dependent on technologies that are currently far beyond reach for many farmers. This does not, however, imply that palliative farming can be rejected out of hand. Opiates, benzodiazepines, and NSAIDs can all be used in escalating doses over several months (as long as the supply is not cut), and for many animals in factory farms, a few months (or weeks) might be all that is needed. These drugs, moreover, are all edible, and therefore do not require any complicated means of administration. It has also been found that chickens that are given the option of choosing feed with and without an NSAID learn to choose an amount of feed containing painkillers that is appropriate to their individual conditions (Danbury et al. 2000).

Indeed, the option of using psychopharmacological interventions on a large scale for prolonged periods of time is mentioned in passing—but is not considered in detail—by Joy A. Mench, a prominent animal behavioral scientist:

As our understanding of the neurochemistry and neurophysiology of fowl increases, it should also be possible to manipulate behavioral and physiological aspects of stress (and underlying emotional states themselves) by administering drugs, receptor agonists and antagonists, or precursors of neurochemicals. For example, the elevated aggression seen in feed-restricted birds during development can be decreased by increasing the dietary level of tryptophan, which is the precursor of serotonin. Serotonin probably not only affects aggression directly, but also has indirect effects because it decreases feeding motivation and thus a part of the stress associated with restriction (Mench 2002: 49).

We do not know if palliative farming can be made technically feasible. While our lack of knowledge is a good reason against taking steps to seek to implement it right away, it is not a good reason against exploring it. On the contrary, it is precisely because it is currently an open question whether palliative farming can be technically feasible that it cannot be rejected out of hand. Given its potential to drastically improve the lives of millions (or billions) of animals in factory farms, and its exclusive dependence on technologies that are already available, we suggest that there is a strong *pro tanto* reason to explore the technical feasibility of palliative farming.

We do, however, concede that there are several ways by which one can object to this conclusion. This will be our focus in the following two sections. In the first section, we consider what we call *principled* objections, according to which palliative farming would not be good *even if* it were technically feasible and could be expected to work according to plan. In the second section, we consider what we refer to as *pragmatic* objections, according to which palliative farming—although it might be good in theory—won’t be technically feasible and/or can be expected to come with larger risks than benefits.

## Principled Objections to Palliative Farming

In considering principled objections to palliative farming, we first consider objections based on concerns about *animal welfare*. Then we turn to objections based on concerns about *animal rights*.

### Animal Welfare

One principled objection to palliative farming could be based on an objection recently voiced by Arianna Ferrari in the context of technological alterations of animals more generally. Ferrari suggests that while such use of technology is most clearly objectionable when it is done for the benefit of humans (e.g., making a dog more likely to win a prize), she also, however, deems it troublesome when done for the sake of animals. The reason, she explains, is that there is a troubling form of paternalism involved in taking for granted that “we humans know best what is good and useful for animals” (Ferrari 2015: 22).

In response to this of objection, it might be argued that palliative farming would not alter the animals if, by *altering animals*, we mean *genetically* altering animals. Nevertheless, it is true that palliative farming aims at bringing about alterations in the neurological processes of animals, and it is also true that any defense of palliative farming rests on the premise that we can know what is good for animals.

Although we should be mindful of the limits to our knowledge, and of our own fallibility, we can almost certainly have knowledge about what is good for animals. To deny this would have radical consequences. If we could not at all know what is good for animals, it is unclear how we could be justified in passing legislation aimed at improving animal welfare, including the EU’s ban on veal crates, barren battery cages, and the tethering of pregnant sows. For such legislation to make sense, we must grant that we can have at least some knowledge about what is good for animals. If we really could not know what is good for animals, we could presumably not have any welfare-based reasons to object to factory farming (or to palliative farming, for that matter). Admittedly, palliative farming might be pursued in ways that *overestimate* our knowledge of what is good for animals. That, however, is a pragmatic concern to which we will return in the next section.

Another principled objection to palliative farming could be that, even if feasible, palliative farming would not be good for animals. But what makes something good, or not good, for animals? What, exactly, constitutes animal welfare? At one level, this depends on specifics about different types of animals at different stages in their lives. The question of what *contributes* to animal welfare is therefore a question for veterinary science. But what *constitutes* a good life for an animal? This is an *evaluative* question, and although this is also a question with which veterinary scientists are concerned, this is not an empirical question, but an ethical one.

So what constitutes animal welfare? Let us first consider the idea of palliative farming in light of two prevalent views.

One view is the *feelings view*, according to which the only thing that matters, ultimately, for an animal's level of welfare is how good it feels to be that animal. Negative feelings such as pain, suffering, frustration, and fear are bad for an animal, and positive feelings, such as pleasure, comfort and contentment are good for the animal (Duncan 2004; Yates 2011; Mellor 2016).

If the feelings view about animal welfare is right, it seems that palliative farming, if effective, could not be ruled out on the ground that, no matter how it was implemented, it would be bad for animals. On the feelings view, palliative farming might, if it could be make to work according to plan, be a major improvement.

Another view is the *preference view*, the idea behind which is that we should not put any restrictions on what ought to be preferred; the realization of whatever is preferred by the individual in question contributes to their welfare (Bruckner 2021). In the case of humans, hedonism (which comes closest to the *feelings view*), and preferentialism can come apart since humans can have preferences that reach well beyond their own time and place. In the case of animals, however, it seems that desire and feeling are very closely linked. Animals, presumably, primarily have desires that concern how things feel to them here and now.

Would animals in factory farms prefer to be drugged? It is difficult to know, since we cannot simply ask animals, which is the reason why veterinary medicine, in contrast to human medicine, does not rely on the informed consent of the patient. It does, however, rely on a veterinarian’s informed judgment about what is in an animal’s interest, and veterinarians routinely make decisions about whether an animal would, all things considered, benefit from drugs that relieve pain and distress.

While animals cannot consent, they can reveal their preferences through their actions. One example is found in the study, cited above, where chickens chose feed with an added NSAID. In considering whether animals in factory farms would prefer to be drugged, it might also be useful to look to Bruce Alexander and colleagues’ famous rat park experiments (Alexander et al. 1979), although we acknowledge that these experiments have been contested (MacBride 2017).^3^ In these experiments, one group of rats was placed in a barren cage and the other group in a ‘rat park’ where they had varied areas for exploration, socialization, and play. The caged group overwhelmingly opted for water with morphine, whereas ‘rat park rats’ were happy with just water, suggesting that the preference of rats for morphine over water is affected by their living conditions. We could, very tentatively, derive from this that factory farmed animals might also prefer ingesting pharmaceuticals that reduce their negative experiences to not having access to those pharmaceuticals, at least in certain circumstances.

The feelings view and preferentialism are, of course, not the only theories of what makes an animal’s life go well. Another theory holds that welfare consists of respecting the animals’ nature (Rollin 1995, 2015). For Rollin, what matters is that we nurture and help fulfil the ‘pigness of pigs’, and the ‘cowness of cows’. We need to protect the interests that constitute an animal’s nature or telos.

One can imagine versions of palliative farming that do, and that do not, respect the animals’ *telos*. Suppose we drug broiler chickens towards the end of their (very short) lives to take the sharpest edge of their suffering when brought to and handled in a slaughterhouse. Perhaps that would still respect the ‘chickennes of chickens’ as it would only change them at the very end of their lives, when they can’t really live according to anything close to their nature anyway. But if we drug a cow to the extent that she no longer cares about whether her newborn calf is taken from her, that will perhaps not respect the ‘cowness of cows’ and might be more problematic on the view that we should respect the animals’ nature.

There are several things to note here. First, one advantage of palliative farming is that, unlike genetic modification, it can be implemented tentatively and in degrees. We can provide different doses of drugs, different kinds of drugs, for different periods of times, at different moments in an animal’s life, and to a particular selection of individuals only. This is an advantage over genetic modification, which is either done, or not done. If we notice that a certain implementation of palliative farming is not in the interests of an animal, we can consider implementing a different version of palliative farming in that same animal.

Another thing to note is that Rollin (2015) is not opposed to modifying animals’ *telos* (e.g., by genetic modification) insofar as we can thereby reduce their suffering. In some situations, this could justify using drugs in a way that changes the animal’s *telos*, if that is the only way (or best available way) to reduce its suffering. Remember that, after all, it was Rollin who suggested in 1995 that we should do research into the genetic bases of animals’ reactions to stressful situations with the aim of breeding animals that are less frustrated by living in cages.

Finally, note that factory-farmed animals are already not living natural lives, but highly restricted lives that are cut short early. Although drugs that dull might have a deleterious effect on the natural functioning of animals in factory farms, it is an open question whether drugs could also, in this context, have a positive effect on natural functioning. Insofar as pain and distress cause aggression, social relations between animals could be better when subjected to palliative farming. As Shriver has pointed out, suffering, in addition to being bad in itself, can be an impediment to realizing important goods (Shriver 2009).

A model of animal welfare widely in use in the animal science literature (albeit not so much in the ethics literature) is the ‘Five Freedoms view’. According to this view, animal welfare is constituted by the fulfillment of the following five measures: (1) Freedom from hunger or thirst; (2) Freedom from discomfort; (3) Freedom from pain, injury or disease; (4) Freedom to express (most) normal behaviour; (5) Freedom from fear and distress (Webster 1994).

This view, arguably, is not (or need not be) a theory about what ultimately constitutes animal welfare, but while this might be regarded as a shortcoming in a philosophical discussion, the view has several virtues. One virtue is that, irrespective of which theory of animal welfare one adopts, one can agree that the Five Freedoms are important (though one can of course accord a different moral weight to each of them). Another virtue is that a common ground can be found, despite different views on what ultimately matters for animal welfare. The Five Freedoms view thus serves a role akin to that of ‘the four principles of biomedical ethics’ (Beauchamp and Childress 2001). A third virtue of the Five Freedoms view is that it is widely institutionalized in studies on animal welfare.

How does palliative farming fare on the Five Freedoms view? It seems that while there could certainly be palliative farming practices that would be opposed to them, there could also be palliative farming practices that are in line with them: practices that give animals better access to freedom from discomfort, freedom from pain, freedom (in some contexts) to express more normal behavior, and freedom from fear and distress. It is therefore difficult to see how an appeal to the Five Freedoms view could make us justified in rejecting palliative farming out of hand.

### Animal Rights

A number of animal ethicists argue that animal welfare (irrespective of which model is employed) is not the only thing that matters in animal ethics. On a rights-based approach to animal ethics (e.g. Regan 1983; Francione and Charlton 2015), animals have a right to not be treated as resources for human exploitation. The right way forward, on such a view, will therefore be to work to end all human exploitation of animals (i.e. abolitionism), rather than to seek to amend a practice that is fundamentally wrongful (i.e. reformism).

Someone who approaches the topic from an animal rights perspective could emphasize that, although palliative farming has some features in common with palliative medicine more broadly, there is nevertheless a major moral difference. When a palliative approach is used in medicine, it is used because there is no course of action available through which the life of the patient can be improved.^4^ In the case of factory farming, by contrast, there *is* in fact another course of action available: to end factory farming. Factory farming is not a natural process that we humans are unable to stop, but is the result of a course of action that we humans, every day, chose to do toward animals. Factory farming is, from an animal rights perspective, an act of wrong-doing toward animals, and this wrongdoing should stop. If, instead, we reform the practice, we are complicit in wrongdoing.

We have two responses. The first response is that it is crucial, if one approaches the issue from an animal rights perspective, to distinguish between the actions of different agents. It might be conceded, on such a view, that it would indeed be wrongful to operate a palliative farm, and also wrongful to buy animal products from palliative farms. It does not follow, however, that it would thereby also be wrong to take measures to ensure that a growing number of those who operate factory farms and buy products from factory farms instead operate palliative farms and buy products from palliative farms. It is not at all clear that it constitutes a rights violation to make rights violations less burdensome for those whose rights are violated.

Our second response is that those who endorse this type of principled animal rights stance would presumably also be committed to opposing passing of the gradually better animal welfare legislation that we have seen, especially in the European Union, over the last decades, such as outlawing hot-iron branding or providing anesthetics to piglets as they get castrated. Our view is that animal welfare measures, such as these, have been steps in the right, not in the wrong, direction.

Admittedly, it might be argued that by pursuing reform, one is like to contribute to delaying the time at which intensive farming practices will be brought to an end. That, however, is not a principled objection, but a pragmatic one, and it is therefore an objection that we will consider in the next section.

## Pragmatic Objections to Palliative Farming

In the previous section we considered *principled* objections to palliative farming. Importantly, however, it is possible to reject a practice even if one does not reject it as a matter of principle. One might argue that, although a practice is not wrongful in principle, the practice might, realistically, be expected to come with so large downsides, compared with its prospective upsides, that it should not be pursued. Such objections, which we refer to as pragmatic objections, are the topic of this section. Are any of these, individually or jointly, sufficiently weighty to justify the conclusion that palliative farming is an option that should not be further pursued?

### Prolonging Intensive Farming Practices

One pragmatic objection is that palliative farming, even if it were found to be technically feasible, would be likely to have the effect of postponing the date at which intensive farming practices are brought to an end. Palliative farming, it might be suggested, communicates that it is legitimate for humans to exploit animals, and by weakening people’s incentives to switch to morally preferable agricultural solutions to feeding humanity.

This is an important worry. Let us first address its communicative part. While we concede that the pursuit of palliative farming would have the effect of legitimizing human exploitation of animals, it is also important to keep in mind that the pursuit of palliative farming communicates that animal welfare matters: it communicates that common factory farming practices are so harsh that the animals in such farms are in urgent need of palliative care. The pursuit of palliative farming would therefore also help to draw attention to the badness of factory farming, and this could spur people into action to improve or abandon current farming practices.^5^ After all, if one holds that palliative farming is abhorrent, then one must presumably hold that standard factory farming is *even more* abhorrent.

Regarding the end of factory farming, we must keep in mind that, on all realistic scenarios, intensive farming practices are not going to be abolished at any time over the coming decades. What will drive most people to move away from consuming factory farmed products, are replacement products that taste as good (or better), are as cheap and are as convenient to purchase as factory farmed products. Palliative farming will not hinder the development of such products.^6^

### Drug Residue in Animal Products

Another pragmatic objection to the pursuit of palliative farming is that this type of farming practice would be likely to have an adverse effect on humans due to drug residue in animal products for human consumption.

In response to this, we would like to concede, first, that the implementation of palliative farming would clearly require protocols that reduce the risk of residue in animal products, and to the extent that there is residue, the residue must be well tolerated by humans.

It is important to keep in mind, however, that we already accept relatively high levels of drug residue in animal products in the case of antimicrobials and deworming agents. There is reason to think that residue from anesthetics is less harmful than residue from antimicrobials. Anesthetics almost entirely dissolve within a few days and do not have negative knock-on effects like antibiotic resistance (Passler 2014). Given the limited amount of animal-based food that most people consume in a day, it is unlikely that, through residue, consumers would receive doses sufficient to cause a significant impact. This is, however, an empirical question that would need to be investigated.

Even if palliative farming would involve some health risk to humans (for a recent discussion of pain-killer residue in livestock, see Steagall et al. 2021), the question remains whether these risks outweigh the significant suffering of billions of animals. Let us say that over time, a heavy meat consumer may, due to the development of tolerance, require a slightly higher dose of painkillers than someone who did not consume meat. That could be a problem; but it could be worth it. It could be that the risks of harm should not be placed only on animals. Even if one thinks that human welfare is far more important than animal welfare, one could still object to letting billions of animals suffer very much for the sake of avoiding a minor health risk to humans (a risk that, moreover, could be avoided by not eating meat produced by factory farming). To take animal suffering seriously in ethics is to accept that sometimes animal interests, if sufficiently large, trump human interests, if sufficiently small. If one denies this, one must ask if one would support a practice that involved increasing the suffering of animals for the sake of a minor improvement in taste. Unless one holds this view, one should welcome measures that redistribute the burdens so that at least some fall onto humans. It may, of course, be that people will not be prepared to eat products from palliative farming because they worry about drug residue (even if there is little reason to be worried). Whether this will be the case is an empirical question. We do know, however, that a growing number of consumers find animal welfare an important consideration when buying animal products. For example, many people prefer to buy meat from pigs that received analgesia during and after castration (Aluwé et al. 2020).

### Drug Stealing

Another concern is that giving large doses of addictive drugs to farm animals could pose a threat to human health because people would try to steal these drugs for human consumption. It would be difficult to protect all industrial farms against theft.

This is a serious problem, but it is one that can be mitigated by administering the drugs via animal fodder. Presumably, animal food production facilities could have a higher level of security than farms, and the drugs could be spread evenly in the food. It would then either be necessary to consume a large amount of animal food to get a noticeable effect from the drugs, or one would have to go through a laborious process of extracting the drug from large quantities of food. It might also be technically possible to make extraction more difficult. This is, however, a legitimate and potentially weighty worry, and we are open to the possibility that, if this problem cannot be effectively overcome, it could constitute a reason against the pursuit of palliative farming.

### Dependence on the Pharmaceutical Industry

A related concern that that must be taken very seriously is if palliative farming would result in a large segment of our economy becoming even more dependent on the pharmaceutical industry. The pharmaceutical industry is already heavily involved in agriculture, most centrally through its widespread use of antibiotics, and we may have good reasons not to create further involvement. A way to seek to mitigate this problem, which ought to be explored, is the option of only using non-patented drugs. However, even if the pharmaceutical industry would become more involved, we would need to weigh this cost against the benefit of reducing suffering in billions of animals.

### Environmental Consequences

The last type of pragmatic concern that we will consider here is the effects of palliative farming on the environment.

We take there to be two distinct environmental concerns. One concern is that drugs and residue from drugs will spill over into the environment, causing pollution. This lies outside of our areas of expertise, but we will note that the environmental risks posed by antibiotics, which are widely used in industrial farming, are in general much more serious than the risks posed by medicines that reduce pain and distress. Both opiates and benzodiazepines are on the US Food and Drug Administration’s “Flush List,” which means that, if not used, they are judged as safe to dispose of by being flushed in the toilet (Food and Drug Administration 2020). A major study from 2017 concludes that in the case of benzodiazepines and opiates, even if all that is produced (legally and illegally) were flushed rather than used, the environmental impact would still be negligible (Kahn et al. 2017). It is possible that the impact of NSAIDs is somewhat larger (Cleuvers 2008). As it would be even more extensive in palliative farming, this would need to be considered. It seems, however, that the risk of environmental damage from drug residue is insufficient to reject the option of palliative farming out of hand.

Another environmental concern is the emission of greenhouse gasses. According to this line of argument, the implementation of palliative farming practices might lead to a comparative increase in the consumption of animal products because people who would otherwise abstain from animal products would, with such farming practices, consume them.

First, to the extent that people’s avoidance of animal products is motivated by health or environmental reasons, we wouldn’t expect them to suddenly start eating products resulting from palliative farming as these wouldn’t be any healthier and would result in at least an equal amount of pollution. But what about those primarily motivated specifically by animal welfare considerations? If they knew that the palliatively farmed animals suffered less, would they increase their consumption of meat, eggs and dairy? We doubt it, especially when they realize that, though the animals’ welfare would be increased, their lives would still be far from optimal.

Finally, note also that not pursuing palliative farming out of concern that people will increase their animal consumption is, in effect, to hold animals hostage by tying greenhouse gas emissions and pollution to their suffering, and to count on their suffering to deter people from contributing to greenhouse gas emissions. If one is willing to continue to inflict suffering on animals in factory farms for the sake of reducing the emission of greenhouse gasses, one should, it seems to us, that one should also be willing to *increase* suffering in factory farms if this could be expected to repel people from buying animal products.

Finally, as Thompson (2021) points out, according to the Food and Agriculture organization (FAO)’s report (Steinfeld et al. 2006), industrial farming practices emit less greenhouse gasses per unit of consumable animal protein that does pasture-based production. So, if the number of farmed livestock remains fixed, then it is better to breed them in industrialized settings than on pasture, at least if reducing greenhouse gas emissions is our priority.

In sum, therefore, it seems that environmental concerns, although they are a type of concern that must be taken very seriously, do not count heavily against the pursuit of palliative farming.

## Conclusion

In this paper we have considered the option of reducing the suffering of factory-farmed animals through the use of drugs that alleviate pain and distress, administered under a regimen where experiential wellbeing is prioritized over the aim of avoiding drug-dependence. Might this be a feasible way to make a significant impact on the poor quality of life of animal in factory farms, by means to a technology that is already developed, tested, and in mass-production?

Our conclusion is that we currently do not know if palliative farming can be made feasible. Based on the considerations that we have explored in this paper, however, the technical feasibility of palliative farming is an issue that we have very strong reasons to investigate. Due to what we take to be the weightiest concerns—the prevention of drug-stealing and environmental damage—it is possible that no justifiable form of palliative farming can in fact be made technically feasible. Whether this is so, however, remains an open question. To determine whether palliative farming is feasible requires interdisciplinary work—in pharmacology, animal welfare science, economics, public policy, etc.—that lies beyond the scope of this paper, and also beyond our own area of research (ethics). Our aim in this paper has not been to say everything that is worth saying, or that it shall constitute a last word in the discussion. On the contrary, our aim has been to help *start* the discussion.

We suggest that palliative farming should be explored as a strategy to reduce animal suffering. It is not proposed as a strategy that should replace existing strategies, such as the promotion of vegetarianism/veganism, or political campaigns for better animal welfare regulations.

We ask that this strategy is assessed by the same standards by which other strategies are assessed. We ask, in other words, that it is assessed in accordance with Neil Levy’s *parity principle*, according to which neurointerventions should be assessed by the same standards of assessment as all other interventions (Levy 2007). We should soberly assess the expected costs and benefits of every proposed intervention, and give neither more nor less weight to an intervention just because it is an intervention that seeks to mitigate the problem by targeting processes in the central nervous system. Sometimes the central nervous system is where the problem lies, and in such cases, this might also be where the solution lies.

## Footnotes

1 Many more fish are farmed, but we restrict ourselves to land animals in this paper.

2 Analgesics are required either legally or by trade organizations in many countries for tail docking and castration in pigs, but often (especially in Europe) the law is often breached. In the US, only 1.1 percent of farms routinely used analgesia or anesthesia when docking tails, and only 5.1 percent of tail-docked cows received analgesia or anesthesia during the procedure (USDA et al. 2018.: i–ii).

3 Thanks to Neil Levy for suggesting to one of the authors that this experiment could be relevant to our paper.

4 Thanks to an anonymous reviewer for bringing our attention to this objection.

5 This is what happened when ethicists defended fulfilling the euthanasia request of a Belgian prisoner. This was partly motivated by the inhumane conditions in the Belgian prison in which he was being detained. Defending his euthanasia request, which caused national and international outrage, spurred the government into action to improve conditions for prisoners (see Devolder 2016).

6 Thanks to an anonymous reviewer for pointing this out.
